# *Clostridium subterminale* Septicemia in a Patient with Metastatic Gastrointestinal Adenocarcinoma

**DOI:** 10.1155/2018/6031510

**Published:** 2018-05-22

**Authors:** Kevin M. Trapani, Leigh J. Boghossian, Elizabeth Caskey

**Affiliations:** ^1^Nazareth Hospital, Philadelphia, PA, USA; ^2^Lankenau Medical Center, Wynnewood, PA, USA; ^3^Philadelphia College of Osteopathic Medicine, Philadelphia, PA, USA

## Abstract

*Clostridium subterminale* is a rare member of the *Clostridiaceae* family that is rarely cultured. This report examines a case of *Clostridium subterminale* cultured from the blood of a 72-year-old man who was ultimately diagnosed with metastatic gastrointestinal (GI) adenocarcinoma. The patient was receiving treatment for nosocomial pneumonia prior to culture of the *C. subterminale*, which led to suspicion for malignancy. Extensive GI and oncologic workup demonstrated multiple comorbidities and a primary GI cancer, which likely caused a breach in the GI mucosa and *C. subterminale* entrance into the bloodstream. After a prolonged intensive care unit (ICU) stay, the patient died on hospital day 23. Though rarely reported, *C. subterminale* septicemia has been demonstrated in patients with malignancy, specifically of the GI tract. Therefore, this case represents a typical *C. subterminale* septicemia patient. Given the prevalence of *Clostridia* and the contemporary emergence of multidrug resistant (MDR) microorganisms, both typical and atypical cases regarding rare members of the species have a significant role in the clinical management and public health planning.

## 1. Introduction

The *Clostridium* genus is comprised of mostly Gram-positive, variably spore-forming anaerobic to aerotolerant rods in pairs or short chains. They most commonly colonize the human GI and female genitourinary tracts, but more rarely, the skin's surface or the oral cavity. They are widely found in the environment, as they are components of the digestive flora in mammals [1]. Given the ubiquity of *Clostridia*, their reservoirs include humans, animals, and the environment. Only a few of the nearly 200 *Clostridia* species are pathogenic to humans, but while they are some of the most extensively studied pathogenic anaerobes, there is limited literature regarding *Clostridium subterminale* [1]. Further, while there are many generalizations regarding *Clostridia*–frequently opportunistic pathogens that produce spores and protein exotoxins–members of the genus exhibit such heterogeneity that there are many exceptions or only specific conditions under which generalizations will apply [1]. Therefore, novel cases, especially of the lesser-studied organisms, are of particular importance among *Clostridium* literature.

## 2. Case Report

A 72-year-old male with a past medical history of quadriplegia, hypertension, hyperlipidemia, neurogenic bladder, type 2 diabetes mellitus, chronic obstructive pulmonary disease, and chronic hepatitis presented to the emergency department with an acute change in mental status. He was a full-time resident at an assisted living nursing home, and his baseline mental status was awake, alert, and oriented to person, place, and time (AAOx3). Upon arrival in the emergency department, he was oriented only to self. Prior to this admission, the patient was being treated for a urinary tract infection and was on day seven of nitrofurantoin and cefepime. On examination, he was hypotensive at a blood pressure of 81/59 mmHg, pulse rate of 115 beats per minute, respiratory rate of 21 breaths per minute, and oxygen saturation of 78% while breathing ambient air. He was in visible respiratory distress and was lethargic, but arousable. He received a nebulizer treatment and was placed on a non-rebreather mask at 6 liters, after which his oxygen saturation rose to 99%. A chest X-ray was taken and revealed left lower lobe pneumonia. His urinalysis was positive for an infection despite current antibiotic compliance. He began vancomycin and piperacillin/tazobactam for health care-associated pneumonia and was admitted to the ICU. Respiratory status worsened, and he required intubation.

Despite these interventions, on his second day of admission, he continued to have copious clear secretions. Pulmonology diagnosed him with aspiration pneumonia and sputum cultures were obtained, which demonstrated moderate white blood cells, rare epithelial cells, few Gram-positive cocci in clusters, and rare yeast. His antibiotics were adjusted for sensitivities to vancomycin and meropenem. To further evaluate his ongoing pulmonary issues, a chest CT scan was obtained, revealing increasing bilateral pleural effusions and patchy sclerotic foci concerning for possible bone metastases, enlarged mediastinal lymph nodes, and nodular infiltrate in the right mid- and upper lung fields. Gastroenterology was consulted for colonoscopy to identify a primary tumor, but the patient was deemed too unstable for a nonemergent procedure. Thoracocentesis revealed exudative pleural effusions negative for malignancy. Abdominal and pelvic CT demonstrated hydronephrosis and a right-sided staghorn renal calculus but no masses.

With these interventions, our patient began to improve, yet daily sedation interruptions and spontaneous breathing trials failed. Initial blood culture results showed Gram-positive bacilli, and further speciation of the organism was requested. On day 13, blood cultures were finalized and grew *Clostridium subterminale*. Sputum and stool testing showed *Pseudomonas aeruginosa* and *Clostridium difficile*, so metronidazole was started. Gastroenterology again stated that the patient was too unstable for colonoscopy and suggested a bone biopsy, which he underwent the following day. The preliminary pathology report stated that the bone biopsy was likely malignant adenocarcinoma, which was confirmed on hospital day 21. Biopsy indicated that the tumor was positive for markers CK7 and CA19-9; primary sites with this immunophenotypic profile include the upper GI tract and pancreaticobiliary system. Carcinoembryonic antigen (CEA) was elevated at 73.8 (normal CEA nonsmokers < 5.1; smokers < 6.6) and CA19-9 was 3,146 (normal < 45). Per oncology, he was not a candidate for any interventions due to his comorbidities.

On day 23, he had increasing secretions and was agitated despite completion of his antibiotic course. The patient's three daughters decided on terminal extubation, and comfort measures were initiated. At 21 : 12 on hospital day 23, the patient was pronounced dead.

## 3. Discussion

In general, the pathogenic species of *Clostridium* are not invasive. As such, available literature has shown that pathogenic *Clostridia* transmission occurs through breaches in the GI tract and wound contamination although spontaneous cases have occurred. Most *Clostridium* species have shown susceptibility to common antibiotics, including penicillin, clindamycin, chloramphenicol, piperacillin, metronidazole, imipenem, and combinations of *β*-lactams with *β*-lactamase inhibitors; others show variable resistance [1]. *Clostridia* also exhibit the ability to form abundant, largely heat-resistant endospores, requiring specific methods for extermination. *Clostridium* toxins can cause a variety of infections, including abscesses, tissue necrosis, and empyema. Symptoms of *Clostridial* septicemia can be vague, often fever, chills, and leukocytosis. The morbidity from *Clostridial* bacteremia is estimated to be 25–50% [1]. Among the *Clostridia* species, *Clostridium difficile*, *Clostridium perfringens*, and *Clostridium botulinum* largely predominate the available literature and reported clinical cases. There are a number of other species notable for infection among the immunocompromised, about which there is little available information. One such species is *Clostridium subterminale*.

In Minelli et al.'s study of normal fecal flora in healthy females, *Clostridia* were found in the intestinal flora of every subject. Of the *Clostridium* species, *C. subterminale* was noted to be one of the most frequently encountered organisms [2]. While this study specifically examined healthy subjects, the authors state, “human flora does not vary greatly from individual to individual,” noting that intestinal flora appears stable in the absence of antimicrobial therapy or disease [2]. In fact, intestinal flora remains as it was established in infancy [3]. Furthermore, *C. subterminale* does not induce gut-associated lymphoid tissue (GALT), one of the GI tract's inherent mechanisms for protection [4]. In light of this, we hypothesize that our patient's comorbidities of GI malignancy and *C. difficile* led to breaches in the colonic mucosa and ultimately *C. subterminale* septicemia. Additionally, his colonoscopy record from 2014 indicated colonic polyps and diverticuli, which are consistent with impaired GI tract integrity. Therefore, *C. subterminale* likely infiltrated his intestinal mucosa, accessing his bloodstream and causing bacteremia.

There is significant variability in disease processes caused by pathogenic *Clostridia*. Furthermore, reliable and efficient identification of *Clostridial* isolates is imperative to establish best management practices. Because of the scarcity of case reports involving *C. subterminale* septicemia, it is worth noting the sources of *C. subterminale* infection. Consequently, many of these sources are from compromised tissue and patients with malignancies, both of which were present in our patient. Sources of *C. subterminale* include but are not limited to abscesses, blood, and wounds. One case report of *C. subterminale* septicemia in a patient with esophageal cancer stated that the likely source of the *C. subterminale* infection was mucosal manipulation during stent placement or repeat endoscopy [5]. Another report described *C. subterminale* septicemia potentially from ulceration near the distal edge of the anal canal in a 51-year-old patient with acute lymphoblastic leukemia [6]. In another case report on *C. subterminale* septicemia in a 41-year-old chronic myelogenous leukemia patient with prior cord blood transplantation, the authors describe the plausibility of previous GI tract colonization by *C. subterminale*, which subsequently accessed the blood though damaged mucosa [7]. In summary, many of the cases of *C. subterminale* septicemia are from patients who were immunosuppressed secondary to malignancy. Additionally, there was evidence of damage to the gastric mucosa in many of these cases, supporting the theory that disruption of the mucosal tissue allowed *C. subterminale'*s entrance into the blood. Due to the frequent association between GI malignancy and *C. subterminale*, we recommend looking for a GI cancer in patients with *C. subterminale* septicemia.

Despite lack of clinical improvement with treatment in our patient, some cases of *C. subterminale* bacteremia recovered. Timely treatment after septicemia identification leads to decreased morbidity and mortality, as well as lower healthcare costs. Given the emergence of MDR organisms and the high frequency of comorbidities and immunosuppression, infection represents a significant cause of morbidity and mortality. Additionally, the cost in disability adjusted life-years from bacteremia and the risk of healthcare-associated infections (HAI's) are significant. In the US, the annual direct costs of HAI's are estimated to be $28.4 to $33.8 billion [8]. In Pennsylvania alone, an independent state agency reported that one year's worth of hospital admissions associated with infections incurred 1,510 additional deaths, 205,000 additional hospital days, and $2 billion in extra hospital charges compared to hospital admissions wherein infections had not occurred [9]. The same agency presented information in their 2012 report that procedures associated with the GI tract had the highest percentage of surgical site infections [9]. *C. subterminale* colonizes the GI tract, and therefore proper management is essential in reducing cost, length of stay, and most importantly, morbidity and mortality. It is clear that efforts to track, prevent, and manage pathogenic bacteria, particularly those that significantly affect immunocompromised and/or hospitalized patients, will represent large improvements to public health in life-years and cost savings.

The significance of the current case report is four-fold: (1) *Clostridium subterminale* is exceedingly rare, and the available literature on the organism is extremely limited; this paper contributes to that important gap in the literature; (2) it demonstrates what we believe is the typical *C. subterminale* septicemia patient; (3) it addresses the traits of *C. subterminale* and the commonalities between *C. subterminale* and a number of other individual *Clostridial* strains; and (4) it provides a compiled source of much of the information available regarding *C. subterminale.* Limitations to this report include the common issue of *Clostridial* misidentification; potential for contamination; and lack of access to this patient's complete medical, social, and family history. Our recommendations for future studies are to document cases of *C. subterminale* using provided and available literature and to further explore its presentation. We hope this will perpetuate the search for avenues to prevent and treat bacteremia, particularly in the immunocompromised with rare microbial infections.
